# Metastatic Jejunal Renal Cell Carcinoma Intussusception Presenting as Melena

**DOI:** 10.7759/cureus.32554

**Published:** 2022-12-15

**Authors:** Emad Elmusa, Muhammad Waleed Raza, Ameer Hamza, Hassan Tahir Khokhar, Mujtaba Butt

**Affiliations:** 1 Internal Medicine, HCA Florida Orange Park Hospital, Orange Park, USA; 2 Gastroenterology, Borland Groover Clinic, Jacksonville, USA

**Keywords:** melena, intussusception, jejunal intussusception, renal cell metastasis, renal cell carcinoma (rcc)

## Abstract

Renal cell carcinoma (RCC) most commonly metastasizes to the lungs, and it is uncommon for RCC to metastasize to the small bowel. Small bowel metastasis commonly presents with gastrointestinal (GI) bleeding. In rare cases, a metastatic small bowel mass can serve as a lead point for intussusception. In this report, we present the case of a male patient whose chief complaint was melena. The patient denied any abdominal pain or nausea. Investigation with push enteroscopy revealed a jejunal mass, and further evaluation with CT showed small bowel intussusception. The patient subsequently underwent small bowel resection and anastomosis. Histopathology confirmed that the jejunal mass was metastatic RCC. We present this case in order to showcase the utility of push enteroscopy in the diagnosis of small bowel metastasis in RCC.

## Introduction

Approximately 90% of renal cell solid tumors turn out to be renal cell carcinoma (RCC) [1]. While RCC most commonly metastasizes to the lungs [1], in 0.7-14.6% of cases, RCC can metastasize to the small bowel [2]. Gastrointestinal (GI) metastasis typically presents as GI bleeding and less commonly as intussusception [1]. Only 30-35% of adult small bowel intussusception cases are secondary to small bowel malignancy [1]. We discuss the case of a patient who denied abdominal pain and whose chief complaint was melena. Investigation with push enteroscopy revealed a jejunal mass. Furthermore, CT showed small bowel intussusception. The patient’s jejunal mass was resected and histopathology revealed that it was RCC metastasis.

## Case presentation

A 75-year-old male with a past medical history significant for stage IV left-sided RCC with metastasis to the lungs status post left nephrectomy on pembrolizumab every three weeks presented with a chief complaint of generalized malaise, fatigue, and melena. The patient reported that approximately a month and a half ago, he had developed melena shortly after starting enoxaparin for bilateral lower extremity deep venous thrombosis. Anticoagulation therapy had been discontinued after the patient developed GI bleeding, but the melena persisted. He had subsequently undergone outpatient esophagogastroduodenoscopy (EGD) and colonoscopy, and both had been non-diagnostic. Due to recurrent melena, he had been scheduled for capsule endoscopy. The patient presented to our institution before completing this.

**Figure 1:**
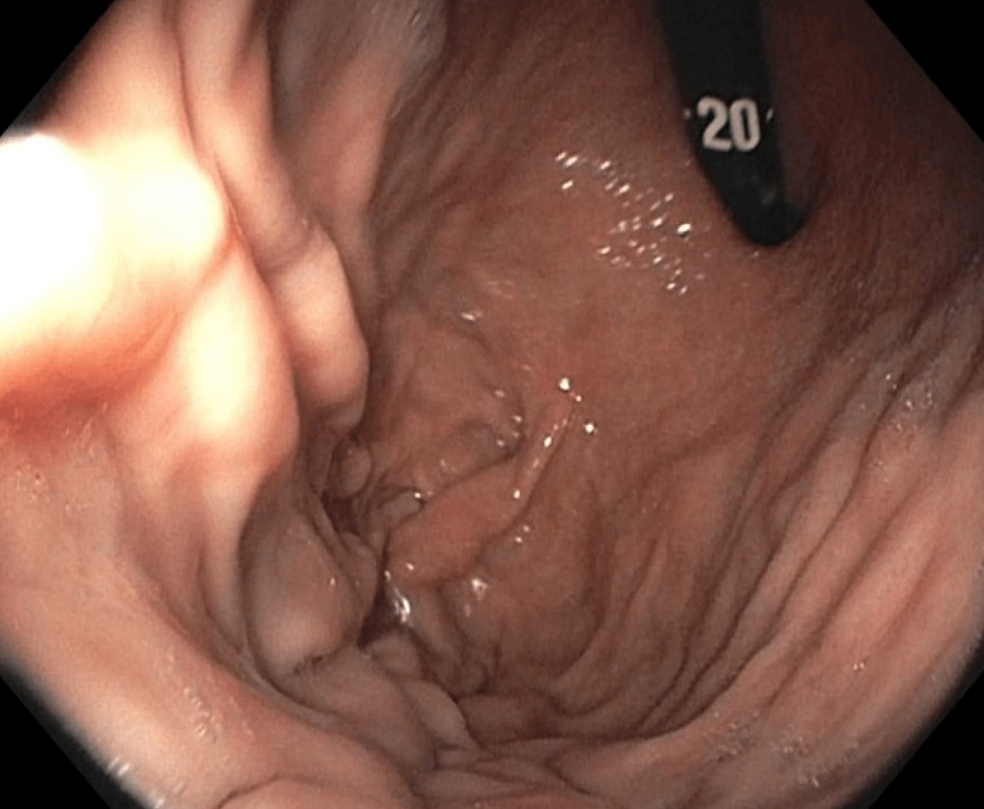
Endoscopic image showing normal gastric mucosa

**Figure 2:**
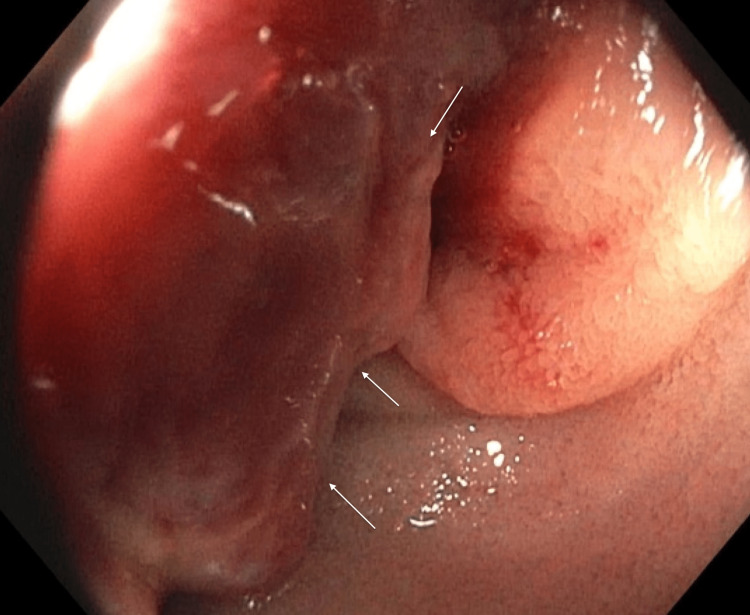
Enteroscopy image showing jejunal mass, fungating appearance

**Figure 3:**
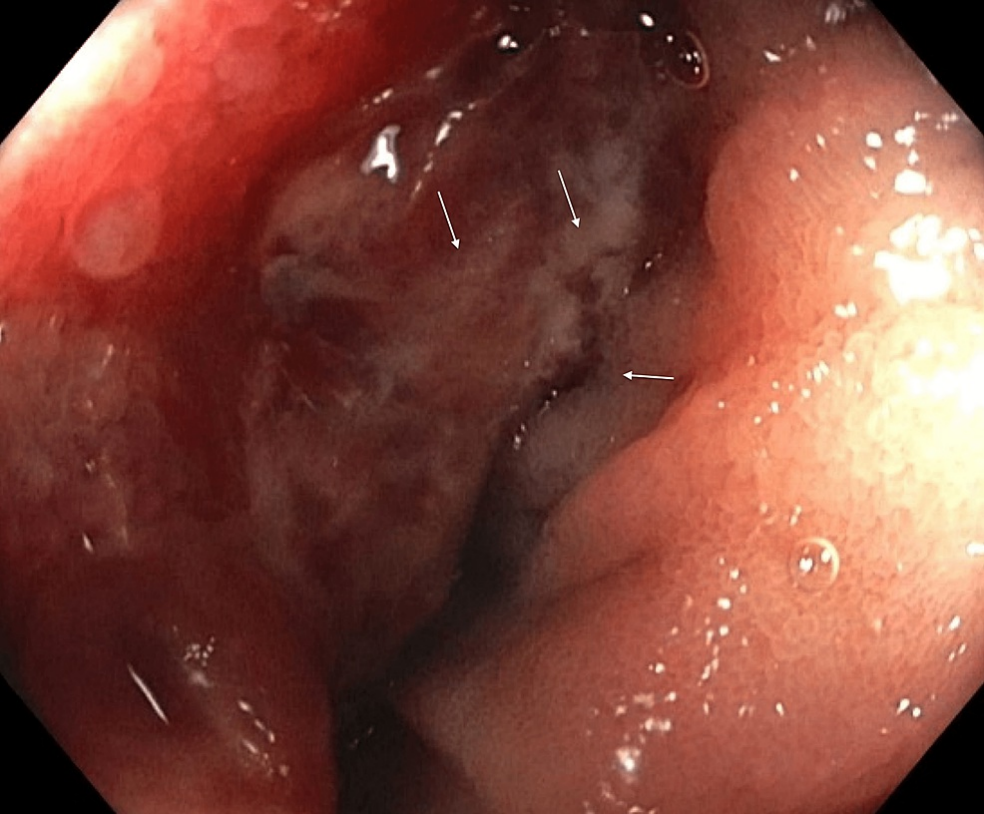
Enteroscopy image showing jejunal mass, ulcerative surface

On presentation in the emergency department, the patient's vitals were as follows: temperature of 36.8 °C, heart rate of 85 bpm, respiratory rate of 16 bpm, blood pressure of 130/76 mmHg, and pulse oxygen saturation of 98%. He denied any abdominal pain or nausea. On physical exam, his abdomen was soft, non-tender, and non-distended. Labs were significant for a hemoglobin of 4.9 g/dL (reference range: 13.7-17.5 g/dL) and a mean corpuscular volume of 79 fl (reference range: 79-92.2 fl). Peripheral intravenous (IV) access was established and the patient was given IV pantoprazole. He was blood-typed and crossmatched, and three units of packed red blood cells were subsequently administered. Gastroenterology took the patient for EGD, which was normal (Figure 1). Push enteroscopy was then performed, which showed a large fungating and ulcerated mass in the jejunum (Figures 2, 3).

**Figure 4:**
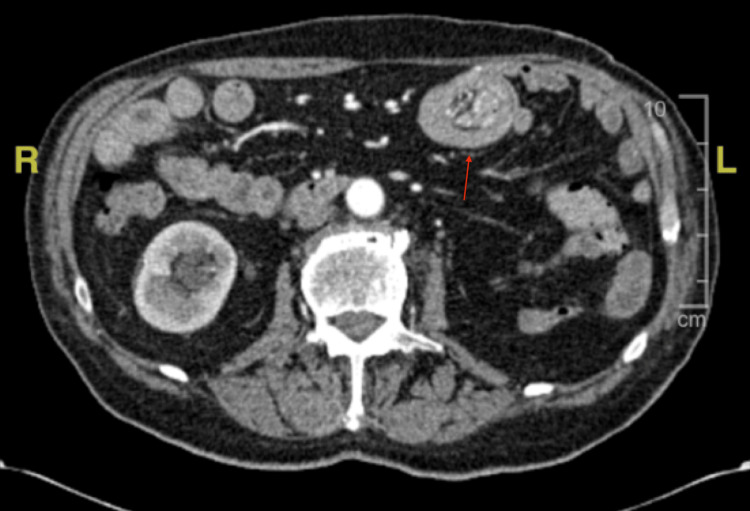
Contrast-enhanced axial CT image demonstrating small bowel intussusception, ‘target sign/donut sign’ CT: computed tomography

**Figure 5:**
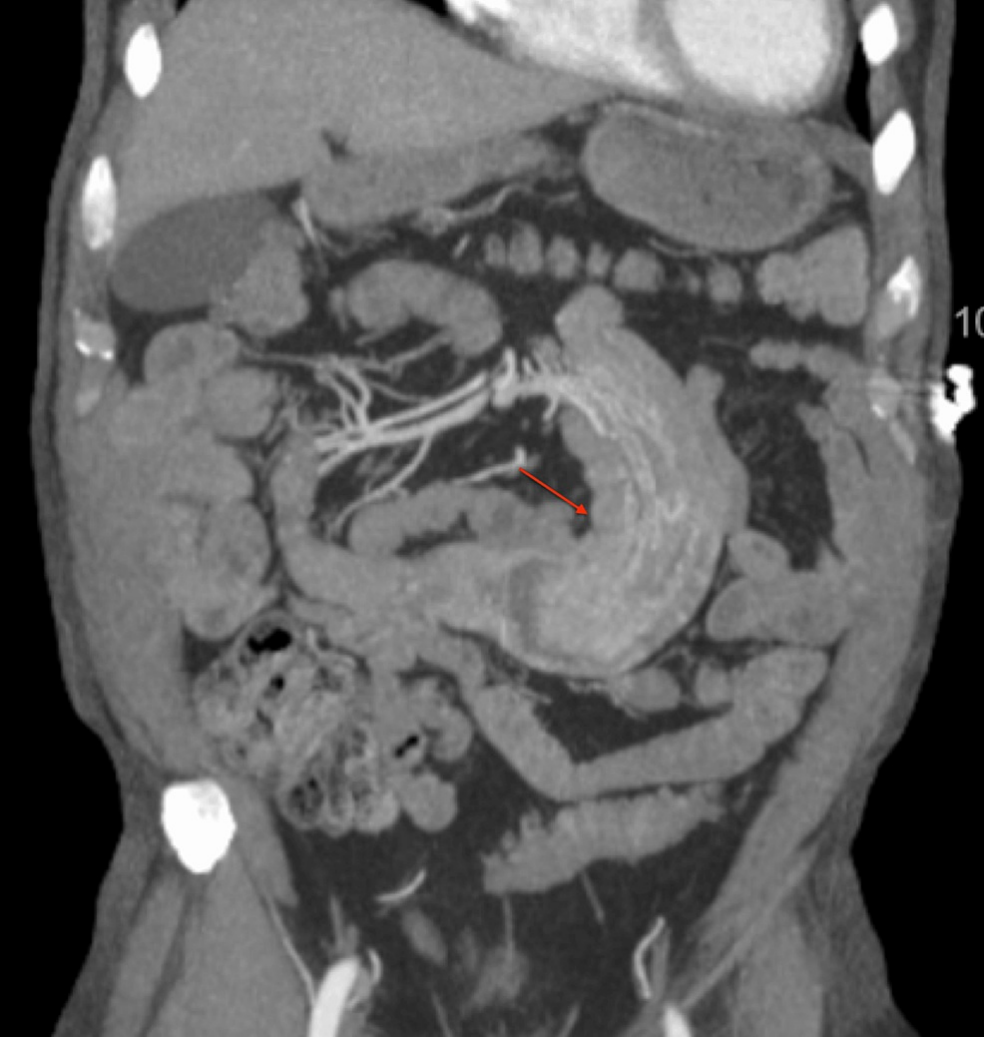
Contrast-enhanced coronal CT image depicting small bowel intussusception CT: computed tomography

A biopsy was taken, and a CT of the abdomen and pelvis with contrast was ordered afterward to evaluate for evidence of other metastatic lesions. It showed small bowel intussusception at the level of the jejunum (Figures 4, 5) and no evidence of other abdominal pelvic masses. General surgery evaluated the patient and recommended surgical bowel resection. The patient wanted to wait for the biopsy results before proceeding with surgical resection. Biopsy showed poorly differentiated carcinoma. Given the patient’s history, this was concerning for metastatic RCC.

**Figure 6:**
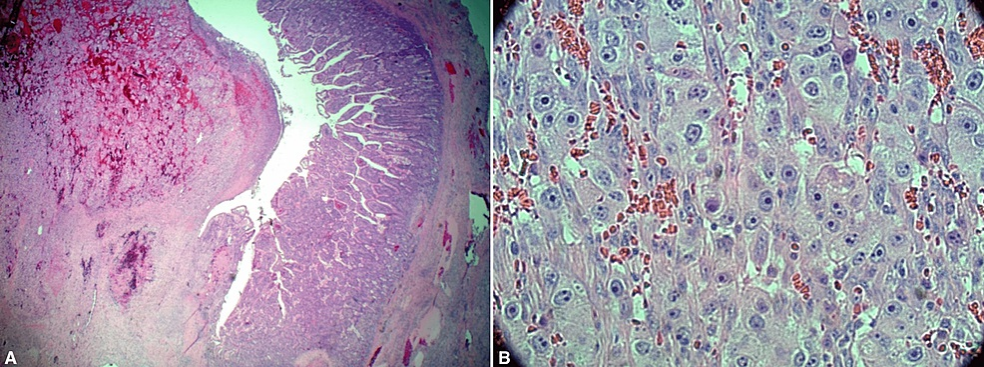
Small bowel resection specimen A: H&E, 2x magnification showing small bowel mucosa on the right and intraluminal tumor on the left; B: H&E, 40x magnification showing features of high-grade RCC RCC: renal cell carcinoma

**Figure 7:**
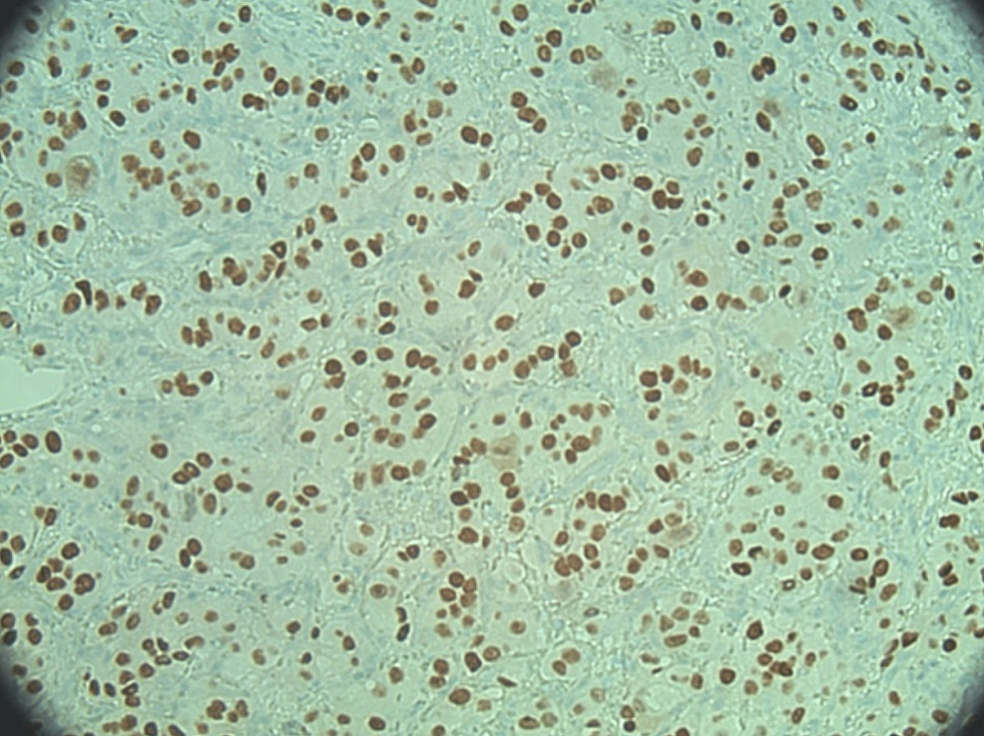
Small bowel resection specimen - PAX-8 immunostain showing positive staining in tumor cells

The patient underwent preoperative evaluation with cardiology, including a left heart catheterization. He had significant coronary artery disease and was considered a high risk for cardiovascular complications intraoperatively. After a discussion regarding risks versus benefits, the patient agreed to undergo resection. He was taken to the operating room for an open small bowel resection. Upon peritoneal incision, the mass was noted within the proximal jejunum, approximately 10 cm from the ligament of Treitz. It was seen to act as a lead point, causing a jejunojejunal intussusception. The intussuscepted bowel was reduced intraoperatively. The jejunal bowel proximal and distal to the mass was resected, followed by small bowel anastomoses. The specimen was sent to pathology. The patient recovered well postoperatively and was discharged on postoperative day five. Histopathology revealed metastatic RCC, extensively involving the small bowel (Figures 6, 7).

## Discussion

Approximately 90% of renal solid tumors are RCC [1]. Cigarette smoking is a risk factor for the development of RCC. At the time of diagnosis, 30-70% of the patients show advanced local or metastatic disease [1]. RCC is capable of hematogenous spread and is historically thought to first metastasize to the lungs via the renal vein and inferior vena cava [3]. Afterward, it can metastasize to any part of the body [3]. RCC can metastasize to the lungs, lymph nodes, liver, bones, brain, adrenal glands, and the contralateral kidney [1]. It metastasizes to the small bowel in 0.7-14.6% of cases [2]. Interestingly, RCC metastasis is more common in post-nephrectomy - at 51% - than in pre-nephrectomy patients - at 24-28% [4]. Additionally, RCC typically metastasizes several years after the initial treatment [4].

The majority of small bowel malignancy is primary, and only 10% of small bowel malignancies are metastatic [1]. Small bowel metastasis more commonly originates from the primary lung, head and neck, breast, esophagus, or melanoma malignancies [1]. Small bowel metastasis typically presents as GI bleeding [5]. Small bowel RCC metastasis rarely presents with obstruction or intussusception [6]. Adult intussusception is far less common compared to the pediatric population, comprising only 5% of total cases [1]. Additionally, of the adult cases that are secondary to malignancy, only 30-35% are within the small bowel [1]. Although ultrasound is the first-line screening tool for the diagnosis of intussusception in children, it is rarely used in adults due to intestinal gas and the widespread availability of CT [7]. The best imaging modality to diagnose intussusception in adults is a CT scan [1,8]. Contrasted studies can assist with assessing the length of the involved segment, localization, and vascular integrity [1]. Surgical resection of the involved bowel is the treatment of choice [1].

Our patient’s chief complaint was melena, which was initially thought to be secondary to anticoagulation therapy. However, the melena persisted even after enoxaparin discontinuation. Upon presentation, the patient was found to be severely anemic, but interestingly, he never complained of abdominal pain and maintained a healthy appetite throughout his hospital course. The patient’s melena and anemia warranted repeat EGD. EGD was unrevealing and push enteroscopy was performed, which revealed a jejunal mass. Duodenum is the least common GI site for metastasis [4]. Therefore, there is utility in performing push enteroscopy in patients suspected to have metastatic masses in the small bowel. The candidates include patients with negative EGD and colonoscopy. Furthermore, push enteroscopy adds additional value over both CT and capsule endoscopy in that biopsy can be performed. Also, our patient was at high risk for cardiovascular events intraoperatively, but because he had a biopsy that supported malignancy, he wished to proceed with surgical resection.

## Conclusions

RCC rarely metastasizes to the small bowel. Moreover, primary or secondary small bowel malignancy is exceedingly rare. Small bowel metastasis commonly presents with GI bleeding and can be a cause of intussusception in rare cases. As demonstrated, our patient’s jejunal mass was only discovered with the aid of push enteroscopy. Biopsy at the time of push enteroscopy revealed that the mass was malignant. We presented this case report to highlight the utility of push enteroscopy in patients with RCC presenting with melena in whom EGD and colonoscopy are unrevealing. This procedure can help expedite diagnosis and treatment planning.
